# Six Letters Addressed to the President of Bethlem Hospital, Respecting the Management of That Establishment, &c.

**Published:** 1842-01

**Authors:** 


Art. II.—*Six Letters addressed to the President of Bethlem Hospital, respecting the Management of that Establishment, &c.* By Philanthropos.—*London*, 1841. 8vo, pp. 28.

These admirable letters are the production, we are told, of a learned Sergeant, a most active and benevolent magistrate of Middlesex. Whoever may be their author, he has good reason to be proud of them; and if the directors of the Institution to which they relate, will only profit by the sound criticisms and enlightened suggestions contained in them, the poor victims of insanity, and medical science, will have equal reason to rejoice at their publication.

"Until recently," says the author, "the proceedings of Bethlem have been a sealed book to the British public. The scanty information contained in the annual statements of the medical officers and the balance-sheets of the auditors afford no aid to the cause of science or humanity. The Report of the Parliamentary Commissioner gave the first insight into the real condition of the Hospital; and the extraordinary interest now excited by the recent reports of the Hanwell Asylum, the great improvements in the treatment of the insane, and some circumstances now before the public connected with the internal discipline of the Hospital itself, renders this a peculiarly fitting opportunity for a calm and temperate enquiry into its real state and condition; an examination into the manner in which the governors discharge their important duties; and the suggestions of alterations and improvement in its management and care."

In his Second Letter, Philanthropos shows that the cures in Bethlem "are comparatively small;" complains that though "the medical staff consists of two physicians, a surgeon, and an apothecary," yet that "the records of the hospital present a disgraceful blank" in everything relating to "statistical knowledge and medical researches." In his Third Letter, the author investigates the subject of *Finance*, and "the result of this investigation establishes the melancholy fact, that with the gross income (including the payments in respect of criminal lunatics),exceeding for the last ten years £20,000 per annum the Hospital has during such period supported only a daily average of 228 patients, possessing no comfort, privilege, or enjoyment, beyond the inmates of a pauper asylum, save the grandeur of the building they inhabit, and the knowledge of the ample remuneration awarded to the officials for protecting their persons and property." In the Fourth Letter, the author enters on the important subject of *Medical Treatment*, and justly complains of the difficulties he experiences in coming to satisfactory conclusions, owing to "the entire absence of all statistical tables, medical journals, and useful reports." He first discusses the great question of *restraint*, and shows satisfactorily that five years ago, at least, (the accessible documents do not permit him to come lower down,) an amount of restraint altogether unjustifiable was in use. "During the year 1836, the total number of curable and incurable patients in the Hospital (I purposely omit the criminal lunatics, because it may be said they are a peculiar class,) was 446; the number of patients at various periods under restraint, 108, being within a fraction one fourth of the whole number; and the number of instances of restraint in the register, 495. The duration of restraints varies as much as the causes; but the censurable negligence with which the restraint-registers have been kept, prevents any statistical tables of periods or causes which can be relied on. This only is clear, that many patients were confined for several days, some for weeks, and it would seem a few for months consecutively."

In the Fifth Letter, Philanthropos examines the subject of the *Present Government of the Hospital,* and shows its extreme faultiness. He "demonstrates that the governors must be quite useless as a controlling power, over the managing part of their own body," while the managing body is shown to be "a mere routine committee for the admission and discharge of patients, and the examination of the hospital provisions;" and that there does not exist any "real, active, searching, superintending power." All this is lamentable; and, accordingly, in his Sixth Letter, the philanthropic author submits to the consideration of the president a list of *Proposed Improvements.* These consist principally, 1, In "the establishment of an independent and unbiassed body of governors;" 2, in the formation of "an active, responsible, representative committee, with ample though defined powers," consisting of from twelve to eighteen members elected annually; 3, "In the appointment of a resident physician, who, for an adequate salary, would be called upon to devote his whole time to the duties of his office." Well may the author say that "this alteration in the management of the Hospital is indispensable."

"This great desideratum once established," continues our author, "all its natural consequences will immediately follow. Aided by some educated mind of superior powers, whose fame and fortune are dependent on his diligence, the committee would proceed, in the adaptation of the Hospital and its inmates, to the vast improvements of which they are susceptible. The gloomy grandeur of the galleries would give way to the simple cheerfulness adapted to the habits of the patients,—the gaol-like windows would be removed,—the costly garden-walls would be demolished,—the airing-grounds planted and rendered cheerful,—the gardens would be opened for the enjoyment of the patients, and the vast space in front of the two wings devoted to their use,—the dietary would be revised, useful journals would be kept, restraints would be repressed, occupations encouraged, and amusements invented—the spirit of intellectual improvement would everywhere be visible; and whilst valuable records for future ages would be yearly accumulating in the annals of the Hospital, the establishment of clinical and other lectures (so successfully introduced into the French asylums) would at once stimulate the resident physician in the performance of his duties, and efface the national stigma that, in this wealthy and benevolent country, we do not possess one school of instruction in this most interesting and important branch of medical education."

In the name of science and humanity we return our thanks to the benevolent and eloquent author of this most seasonable appeal; and we confess that we look forward with considerable confidence to the early advent of the time when this establishment shall be as conspicuous in its moral and scientific relations as it now is in its physical and financial features; for we entirely coincide with the author of this pamphlet in believing that "a perusal of the list of governors is alone sufficient to satisfy the most prejudicial objector, that no abuse will be wilfully upheld; and that if Bethlem does not take that rank in public estimation to which, by its wealth, its power, and its respectability, it is entitled, it is because of the sluggish nature of its constitution, and the unhesitating belief of its members that all is rightly done when all is rightly intended."

